# The relationship between internet addiction and risk of suboptimal health status among Chinese college students

**DOI:** 10.1097/MD.0000000000034528

**Published:** 2023-08-18

**Authors:** Chunxiao Ma, Zhongyu Ren, Caifu Li

**Affiliations:** a School of Business Management, Liaoning Technical University, Liaoning, China; b Research Institute of Educational Economics and Administration, Shenyang Normal University, Liaoning, China; c School of Physical Education, Southwest University, Chongqing, China; d College of Sports Science, Shenyang Normal University, Liaoning, China.

**Keywords:** cross-sectional study, internet addiction, suboptimal health status

## Abstract

Internet addiction (IA) is a prevalent trend among college students, and the relationship between severe IA and poor health status among college students has been well established. However, whether IA is associated with suboptimal health status (SHS) in college students is unclear. This study aimed to investigate the association between IA and SHS risk in Chinese college students. We conducted a cross-sectional study to assess whether IA was related to SHS risk in 2265 college students in Shenyang, China. SHS was assessed using the Suboptimal Health Status Questionnaire with a cutoff score of ≥35 to document SHS. IA was assessed using the validated 20-item Young’s Internet Addiction Test with cutoff scores of 31–49 and 50–100 for mile and moderate-to-severe cases, respectively. The prevalence rate of SHS was 54.0%. After adjusting for potential confounding factors, the IA categories were positively related to a higher risk of SHS. The odds ratios (95% confidence intervals) for SHS across IA categories were 1.00, 7.66 (6.00, 9.78), and 27.93 (20.95, 37.24) (*P* for trend: <.001) after adjusting for multiple confounding factors. This is the first cross-sectional study to demonstrate that IA is independently associated with SHS. This finding suggests that IA is a negative risk factor for SHS.

## 1. Introduction

Suboptimal health status (SHS) is considered a stress-induced intermediate state between health and disease, which is characterized by the patient’s perception of their health complaints, general weakness, and low energy within a period of 3 months,^[1]^ and is a subclinical, reversible stage of chronic disease.^[2]^ A nationally representative study conducted in Sweden reported an SHS prevalence of 20.5%.^[3]^ Compared with Western populations, the prevalence of SHS in China is between 46.0%^[4]^ and 55.9%.^[5]^ SHS is a high-risk factor for cardiovascular disease^[6]^ and type 2 diabetes mellitus.^[7]^ Therefore, as a preventive prerequisite, the identification of factors that contribute to SHS is urgently required.

Recently, a negative and trending social phenomenon, internet addiction (IA), which is defined as a new clinical disorder characterized by difficulty in controlling one’s internet usage,^[8]^ has become a serious social problem and is showing a progressively higher prevalence. For example, a meta-analysis of 31 countries across 7 world regions showed that 6.0% of global participants were addicted to the internet.^[9]^ Compared with these countries, the Chinese population has a higher prevalence of IA, especially among college students, with a prevalence rate of 11.7%. Interestingly, IA may have a negative effect on physical (i.e., higher cortisol levels,^[10]^ abnormal heart rate variability,^[11]^ and lower respiratory sinus arrhythmia^[12]^) and mental wellbeing (i.e., depression,^[13]^ anxiety,^[13]^ and poor sleep quality^[14]^). Considering the threshold for disease prevention, exploring the association between IA and SHS is essential. To examine the association between IA and SHS and add new evidence to the research field, we conducted a cross-sectional study among Chinese college students.

## 2. Materials and methods

### 2.1. Participants

The source data for this study were collected in 2022. This study was conducted as part of the “adolescent health-promoting program,” which was conducted by 4 universities in Shenyang (i.e., Shenyang Normal University, Liaoning University, Shenyang Institute of Engineering, and Shenyang University of Technology). We invited 2300 college students to participate in the annual physical fitness examination; of these, 2265 agreed to participate in the survey and provided informed consent. The study protocol was approved by the Institutional Review Board of the Research Institute of Educational Economics and Administration of Shenyang Normal University.

### 2.2. Measures

#### 2.2.1. Assessment of IA.

A validated Chinese version of Young’s Internet Addiction Test was used to assess IA.^[15]^ The Internet Addiction Test is comprised of 20 items, with responses ranging from (1) “not at all” to (5) “always” and a total score ranging from 20 to 100. A high IA score indicated a more severe level of IA. IA was classified using the following cutoff values: normal (0–30 points), mild (31–49 points), and moderate-to-severe (50–100 points). Our IA reliability analysis revealed a Cronbach’s alpha of 0.961. This indicates that the IA had strong internal consistency.

#### 2.2.2. Evaluation of SHS.

SHS was evaluated using the Suboptimal Health Status Questionnaire (SHSQ-25),^[1]^ which has 25 items and responses ranging from (1) “never or almost never” to (5) “always” on a 5-point Likert scale. Raw scores of 1 to 5 on the questionnaire were recorded as 0 to 4 points. The total scores produced by all 25 items ranged from 0 to 100, with higher values indicating greater severity. A cutoff value (SHSQ score ≥ 35) was used to distinguish whether the participants had ideal health status or SHS.^[16]^ Cronbach’s α coefficient was used to describe the reliability of the Chinese version of the SHS. The reliability analysis revealed a Cronbach’s alpha coefficient for the SHSQ-25 of 0.978, indicating that the SHSQ-25 used in this study possessed excellent internal consistency.

#### 2.2.3. Relevant covariates.

Anthropometric variables (height and body weight) were measured using the standard protocols. Body mass index (BMI) was calculated as weight (kg)/height^2^ (m^2^). Demographic variables and lifestyle factors, including sex, age (continuous variable), residence (urban or rural), smoking status (never, ex-smoker, sometimes, or everyday), drinking status (never, ex-drinker, sometimes, or everyday), and sleep quality (good or bad), were assessed via a self-administered questionnaire.

### 2.3. Statistical analysis

All statistical analyses were performed using Stata software (Stata Corp LP, College Station, TX, USA). All continuous and categorical variables were presented as means (95% confidence interval [CI]) and percentages). Differences in the subject characteristics between IA severity levels were examined using analysis of variance for continuous variables and the chi-square test for proportional variables.

Analysis of covariance analysis was used to evaluate the differences in SHS scores between categories of IA severity after adjusting for sex, age, BMI, residence, smoking status, drinking status, and sleep quality. IA level and SHS were used as the independent and dependent variables, respectively. Multiple logistic regression analysis was used to examine the association between IA and SHS risk after adjusting for relevant covariates. *P* < .05 was considered statistically significant in all 2-sided tests.

## 3. Results

The participant characteristics according to IA categories are presented in Table 1. Participants with severe IA were more likely to be male, reside in rural areas, have a higher drinking status (sometimes), and have poorer sleep quality (All *P* value: <.001). We observed no significant association between the IA categories and other participant characteristics.

**Table 1:** Participants’ characteristics according to IA category.*

N = 2265	IA category			*P* value†
	Normal	Mild	Moderate and severe	
	20.0–30.0	31.0–49.0	50.0–100.0	
Sex (male), %	68.3	60.7	68.6	.001
Age, yr	18.7 (18.6, 18.8)	18.7 (18.6, 18.8)	18.8 (18.7, 18.9)	.189
BMI, kg/m^2^	19.7 (19.5, 19.9)	19.6 (19.4, 19.8)	19.7 (19.5, 19.9)	.693
Residence (urban), %	50.0	52.8	45.8	.023
Smoking status, %				
Smokers	1.2	0.9	1.1	.844
Ex-smokers	32.1	32.5	33.3	.884
nonsmokers	66.7	66.7	65.5	.876
Drinking frequency, %				
Everyday drinkers	1.2	0.4	1.3	.100
Sometimes drinkers	32.1	34.1	38.6	.032
Nondrinkers	64.7	64.4	58.1	.013
Ex-drinkers	2.0	1.1	2.1	.247
Poor sleep quality, %	10.7	15.2	20.9	<.001

BMI = body mass index, SD = standard deviation.

* Continuous and categorical variables are expressed as mean (95% confidence interval) or percentages.

† *P* value was obtained using ANOVA in both continuous or chi-square test for categorical variables among IA category.

In this study, we adjusted for sex, age, BMI, residence, smoking status, drinking status, and sleep quality. The results showed that SHS scores differed significantly among the IA categories. The mean and 95% CI for normal, mild, and moderate-to-severe were 26.7 (26.0, 27.4), 38.2 (37.4, 39.0), and 65.4 (64.7, 66.1), respectively (*P* for trend: <.001) (Table 2).

**Table 2:** Multivariable-adjusted association between IA category and SHS level.

	Low level	Middle level	High level	*P* for trend*
No. of participants	756	801	708	–
Crude Model 1†	31.0 (29.8, 32.3)	42.5 (41.4, 43.7)	58.5 (57.2, 59.7)	<.001
Adjusted Model 2†	31.2 (30.0, 32.3)	42.4 (41.2, 43.6)	58.4 (57.2, 59.6)	<.001
Adjusted Model 3†	31.0 (29.8, 32.2)	42.7 (41.5, 43.8)	58.3 (57.1, 59.5)	<.001
Adjusted Model 4†	26.7 (26.0, 27.4)	38.2 (37.4, 39.0)	65.4 (64.7, 66.1)	<.001

IA = internet addiction, SHS = suboptimal health status.

* Linear trend was obtained by using ANCOVA for SHS score.

† Four different logistic regression models were used: model 1: crude; model 2: adjustment for sex, age; model 3: body mass index, residence (urban or not), smoking status (never, ex-smoker, sometimes, or everyday), drinking status (never, ex-drinker, sometimes, or everyday); model 4: sleep quality.

The prevalence of SHS was 54.0% (1224/2265). We analyzed the adjusted relationships between IA categories and SHS risk. In the adjusted model, the IA categories were positively related to a higher risk of SHS. The odds ratios (95% CI) for SHS across IA categories were 1.00, 7.66 (6.00, 9.78), and 27.93 (20.95, 37.24) (*P* for trend: <.001) after adjusting for multiple confounding factors (Table 3).

**Table 3:** Adjusted associations between IA category and risk of SHS.

	Low level	Middle level	High level	P for trend*
No. of participants	756	801	708	–
No. of SHS (SHS score ≧ 35)	129	492	603	–
Crude OR (95% CI), Model 1†	Reference	7.74 (6.11, 9.81)	27.91 (21.08, 36.96)	<.001
Adjusted OR (95% CI), Model 2†	Reference	7.70 (6.06, 9.77)	29.01 (21.84, 38.53)	<.001
Adjusted OR (95% CI), Model 3†	Reference	7.82 (6.15, 9.94)	29.21 (21.96, 38.86)	<.001
Adjusted OR (95% CI), Model 4†	Reference	7.66 (6.00, 9.78)	27.93 (20.95, 37.24)	<.001

CI = confidence interval, IA = internet addiction, OR = odds ratio, SHS = suboptimal health status.

* Linear trend was obtained by using multiple logistic regression analysis.

† Four different logistic regression models were used: model 1: crude; model 2: adjustment for sex, age; model 3: body mass index, residence (urban or not), smoking status (never, ex-smoker, sometimes, or everyday), drinking status (never, ex-drinker, sometimes, or everyday); model 4: sleep quality.

## 4. Discussion

We aimed to examine the relationship between IA and SHS risk, and demonstrated that IA was significantly associated with a higher risk of SHS. This is the first study to evaluate the relationship between IA and SHS among Chinese college students.

Currently, the relationship between a healthy lifestyle (i.e., spiritual growth, health responsibility, physical activity, nutrition, interpersonal relationships, and stress management) and increased risk of SHS is widely accepted.^[4,5]^ Regarding specific life habits, whether IA is associated with SHS remains unclear. Our results suggest that IA is an independent indicator of SHS.

Regarding the potential mechanisms for the negative effects of IA on the development of SHS, enhanced oxidative stress may play an important role in the association between IA and SHS. A previous study demonstrated that severe IA may lead to increased leukocyte telomere length as a result of oxidative stress.^[17]^ Oxidative stress can induce lipid peroxidation, protein oxidation, and DNA damage, leading to abnormal cell structure and an increased risk of SHS.^[18]^ Additionally, IA may induce the development of SHS through the bright light emitted by computer/mobile devices, which suppresses stress-related melatonin secretion.^[19]^ In general, when people are alone, such as during meals, while riding the bus, or before going to sleep, they prefer entertainment activities on their smartphones (e.g., reading gossip and news, playing games, or chatting with friends).^[20]^ The light emitted by computer or mobile devices with light-emitting diode displays is strong. Light exposure, especially high-intensity light delivered across the eyelids using an over-eye mask suppressed serum melatonin levels and delay dim light melatonin onset.^[21]^ Another plausible reason for the findings of this study is sleep quality. Our finding that students with severer IA tend to have poorer sleep quality is similar to findings of previous studies.^[22]^ Poor sleep quality may increase the stress resulting from sleep deprivation.^[23]^ However, these results remained significant after adjusting for sleep quality. Thus, we conclude that the significant relationship between IA and SHS risk is independent of sleep quality. Furthermore, other potential confounding factors that could influence the results were analyzed, including sex, age, BMI, residence, smoking status, and drinking status. A significant association between IA and SHS risk remained even after adjusting for these potential confounders, indicating that IA is independently associated with SHS.

The present study has several limitations. First, this was a cross-sectional study; therefore, the causality between IA and SHS could not be established. Second, our study results are not representative of the general population; therefore, further investigations with larger sample sizes are required to confirm our findings. Third, although our study adjusted for confounding factors, other important factors that affect the association between IA and SHS might exist.

## 5. Conclusions

This cross-sectional study revealed a significant relationship between IA and high SHS risk. IA may promote SHS development among Chinese college students. Further prospective and interventional studies are needed to confirm this association.

## Acknowledgments

We would like to thank the all college students who gave their informed consent and agreed to participate in this study. We would also like to thank our staff for their dedicated work.

## Author contributions

**Conceptualization:** Chunxiao Ma, Caifu Li.

**Data curation:** Chunxiao Ma, Caifu Li, Zhongyu Ren.

**Formal analysis:** Chunxiao Ma, Caifu Li, Zhongyu Ren.

**Investigation:** Chunxiao Ma, Caifu Li.

**Methodology:** Chunxiao Ma, Caifu Li.

**Writing – original draft:** Chunxiao Ma, Zhongyu Ren.

**Writing – review & editing:** Chunxiao Ma, Caifu Li.

## References

[1] Yan Y-X Liu Y-Q Li M. Development and evaluation of a questionnaire for measuring suboptimal health status in urban Chinese. J Epidemiol. 2009;19:333–41. 19749497 10.2188/jea.JE20080086 PMC3924103

[2] Wang W Yan Y. Suboptimal health: a new health dimension for translational medicine. Clin Transl Med. 2012;1:1–6. 23369267 10.1186/2001-1326-1-28 PMC3561061

[3] Taloyan M Aronsson G Leineweber C. Sickness presenteeism predicts suboptimal self-rated health and sickness absence: a nationally representative study of the Swedish working population. PLoS One. 2012;7:e44721. 22984547 10.1371/journal.pone.0044721 PMC3439368

[4] Chen J Cheng J Liu Y. Associations between breakfast eating habits and health-promoting lifestyle, suboptimal health status in Southern China: a population based, cross sectional study. J Transl Med. 2014;12:348. 25496597 10.1186/s12967-014-0348-1 PMC4269950

[5] Bi J Huang Y Xiao Y. Association of lifestyle factors and suboptimal health status: a cross-sectional study of Chinese students. BMJ Open. 2014;4:e005156. 10.1136/bmjopen-2014-005156 PMC4067885 24951109

[6] Yan YX Dong J Liu YQ. Association of suboptimal health status and cardiovascular risk factors in urban Chinese workers. J Urban Health. 2012;89:329–38. 22203493 10.1007/s11524-011-9636-8 PMC3324604

[7] Ge S Xu X Zhang J. Suboptimal health status as an independent risk factor for type 2 diabetes mellitus in a community-based cohort: the China suboptimal health cohort study. EPMA J. 2019;10:65–72. 30984315 10.1007/s13167-019-0159-9 PMC6459451

[8] Young KS. Internet addiction: the emergence of a new clinical disorder. Cyberpsychol Behav. 1998;1:237–44.

[9] Cheng C Li AY-l. Internet addiction prevalence and quality of (real) life: a meta-analysis of 31 nations across seven world regions. Cyberpsychol Behav Soc Netw. 2014;17:755–60. 25489876 10.1089/cyber.2014.0317 PMC4267764

[10] Kim EH, 김대란. Comparison of stress level and HPA axis activity of internet game addiction vs. Non-addiction in adolescents. J Korean Biol Nurs Sci. 2013;15:173–83.

[11] Kim N Hughes TL Park CG. Altered autonomic functions and distressed personality traits in male adolescents with internet gaming addiction. Cyberpsychol Behav Soc Netw. 2016;19:667–73. 27831751 10.1089/cyber.2016.0282

[12] Lee JY Shin KM Cho S-M. Psychosocial risk factors associated with internet addiction in Korea. Psychiatry Investig. 2014;11:380–6. 10.4306/pi.2014.11.4.380 PMC4225201 25395968

[13] Younes F Halawi G Jabbour H. Internet addiction and relationships with insomnia, anxiety, depression, stress and self-esteemin university students: a cross-sectional designed study. PLoS One. 2016;11:1–13. 10.1371/journal.pone.0161126 PMC5019372 27618306

[14] Zhang MWB Bach Xuan T Le Thi H. Internet addiction and sleep quality among Vietnamese youths. Asian J Psychiatry. 2017;28:15–20. 10.1016/j.ajp.2017.03.025 28784371

[15] Wu X-S Zhang Z-H Zhao F. Prevalence of Internet addiction and its association with social support and other related factors among adolescents in China. J Adolesc. 2016;52:103–11. 27544491 10.1016/j.adolescence.2016.07.012

[16] Hou H Feng X Li Y. Suboptimal health status and psychological symptoms among Chinese college students: a perspective of predictive, preventive and personalised health. EPMA J. 2018;9:367–77. 30538788 10.1007/s13167-018-0148-4 PMC6261912

[17] Kim N Sung JY Park JY. Association between internet gaming addiction and leukocyte telomere length in Korean male adolescents. Soc Sci Med. 2019;222:84–90. 30616218 10.1016/j.socscimed.2018.12.026

[18] Spencer WA Jeyabalan J Kichambre S. Oxidatively generated DNA damage after Cu(II) catalysis of dopamine and related catecholamine neurotransmitters and neurotoxins: role of reactive oxygen species. Free Radic Biol Med. 2011;50:139–47. 21075203 10.1016/j.freeradbiomed.2010.10.693 PMC3353411

[19] Czeisler CA Shanahan TL Klerman EB. Suppression of melatonin secretion in some blind patients by exposure to bright light. N Engl J Med. 1995;332:6–11. 7990870 10.1056/NEJM199501053320102

[20] Enez Darcin A Kose S Noyan CO. Smartphone addiction and its relationship with social anxiety and loneliness. Behav Inf Technol. 2016;35:520–5.

[21] Figueiro MG Rea MS. Preliminary evidence that light through the eyelids can suppress melatonin and phase shift dim light melatonin onset. BMC Res Notes. 2012;5:221. 22564396 10.1186/1756-0500-5-221 PMC3469368

[22] Demirci K Akgönül M Akpinar A. Relationship of smartphone use severity with sleep quality, depression, and anxiety in university students. J Behav Addict. 2015;4:85–92. 26132913 10.1556/2006.4.2015.010 PMC4500888

[23] Ahrberg K Dresler M Niedermaier S. The interaction between sleep quality and academic performance. J Psychiatr Res. 2012;46:1618–22. 23040161 10.1016/j.jpsychires.2012.09.008

